# Science Highlights and Final Updates from 17 Years of Total Solar Irradiance Measurements from the *SOlar Radiation and Climate Experiment*/*Total Irradiance Monitor* (SORCE/TIM)

**DOI:** 10.1007/s11207-021-01853-x

**Published:** 2021-09-03

**Authors:** Greg Kopp

**Affiliations:** Laboratory for Atmospheric and Space Physics, Univ. of Colorado, Boulder, CO 80303 USA

**Keywords:** Solar irradiance, Solar variability, Earth climate, Solar cycle, Integrated Sun observations

## Abstract

The final version (V.19) of the total solar irradiance data from the *SOlar Radiation and Climate Experiment* (SORCE) *Total Irradiance Monitor* has been released. This version includes all calibrations updated to the end of the mission and provides irradiance data from 25 February 2003 through 25 February 2020. These final calibrations are presented along with the resulting final data products. An overview of the on-orbit operations timeline is provided as well as the associated changes in the time-dependent uncertainties. Scientific highlights from the instrument are also presented. These include the establishment of a new, lower TSI value; accuracy improvements to other TSI instruments via a new calibration facility; the lowest on-orbit noise (for high sensitivity to solar variability) of any TSI instrument; the best inherent stability of any on-orbit TSI instrument; a lengthy (17-year) measurement record benefitting from these stable, low-noise measurements; the first reported detection of a solar flare in TSI; and observations of two Venus transits and four Mercury transits.

## Introduction

The total solar irradiance (TSI) is the spatially and spectrally integrated radiant energy from the Sun at a distance of one astronomical unit. Recognized for its importance in Earth-climate studies (Lean, 1997, 2010, 2017; Lean et al., 2005; Lean and Rind, 2008; Gray et al., 2010; Haigh, 2011; IPCC, 2013; Solanki, Krivova, and Haigh, 2013; Jungclaus et al., 2017; Matthes et al., 2017), the TSI has been measured from space via a series of overlapping missions from NASA, ESA, and NOAA since 1978. The TSI is the primary energy input to the Earth’s climate system, being 3000 times larger than all other energy sources combined (Kren, Pilewskie, and Coddington, 2017). This energy varies with time, having $\approx10^{-10}~\text{year}^{-1}$ variability due to stellar evolution on billion-year timescales, $\approx0.1\%$ variability over the course of the 11-year solar cycle, up to 0.3% variability on solar-rotational (27-day) timescales, 0.01% variations on five- to ten-minute timescales, and estimated variations of $\approx0.05\%$ on century timescales (Kopp, 2014). This latter value is the least certain due to the relatively short measurement record; yet it is the most relevant for climate studies.

While still employing the same fundamental concepts of electrical substitution radiometry as all other TSI instruments, the *Total Irradiance Monitor* (TIM) onboard the *Solar Radiation and Climate Experiment* (SORCE) mission (Rottman, 2005) introduced several new instrument improvements described by Kopp and Lawrence (2005) and briefly summarized here:

i) This was the first instrument to place the precision aperture, which accurately determines the area over which the incident sunlight is collected, at the front of the instrument instead of deep inside, reducing scatter.
ii) The use of diffuse metallic nickel–phosphorus instead of black paint as the absorptive material inside the radiometers provides improved thermal conductivity and better stability from damaging ultraviolet and harder solar radiation, giving the TIM the best inherent stability of any on-orbit TSI instrument.
iii) Phase-sensitive-detection approaches to operating the instrument’s internal servo-control systems and to analyzing the resulting data in ground processing provide lower noise than all other instruments (Kopp, 2014).
iv) The TIM is the first TSI instrument to employ a digital signal processor for servo-system control, enabling use of PID control loops.
v) A feed-forward system anticipates changes in radiometer electrical power needed as a shutter modulates incoming sunlight, thus reducing the radiometer’s servo error signal and allowing better control-system stability.
vi) Pulse-width modulation of a very stable 7.1-V DC reference voltage applies power linearly.

Many of these instrument improvements resulted in TSI-record measurement accuracy and stability improvements. Most notable is the TIM’s establishment of the now-accepted, lower TSI value than that measured prior to the SORCE launch (Kopp, Lawrence, and Rottman, 2005; Kopp and Lean, 2011). This value is now the IAU-accepted solar-irradiance value (Prša et al., 2016) and has helped reduce differences between measured incoming and outgoing radiation (Loeb et al., 2012; L’Ecuyer et al., 2015; Loeb and NCAR Staff, 2018) to better understand the Earth’s radiation imbalance. This article presents the primary scientific highlights, such as this, of the TIM’s 17-year measurement record (Section 4) and gives an overview of the final-data version’s calibrations (Section 3) and operational changes necessitated during the SORCE mission lifetime (Section 2). Details of the instrument design and functionality are not described; for those, see Kopp and Lawrence (2005) and Kopp, Heuerman, and Lawrence (2005).

## Operations Overview

The TIM observed the Sun during the daylight portion of almost all of the SORCE spacecraft’s 605-km, 95-minute-period, low-Earth orbits. In the nominal phase of the mission, dark measurements (Section 3.3) were acquired during the eclipsed portions of the orbits. Over time, these were fitted to relevant instrument temperatures (as functions of $T^{4}$) to provide a thermal model used to estimate the background (thermal) contributions to the signal for instrument temperatures appropriate for the solar-viewing orbit portions. Calibrations of the instrument servo-system gain (Section 3.2) were acquired monthly, requiring six-hour continuous blocks of time. Intermittent comparisons between the four relatively independent TIM cavities allowed tracking of degradation in the primary cavity using duty cycles of 1%, 0.5%, and 0.25% for the other cavities (Section 3.1).

### Power Cycling

As the spacecraft aged and the batteries degraded, instrument operations during the eclipsed portions of the orbits were restricted. Deemed the highest-priority instrument at the time, the TIM was the last of the four SORCE instruments to continue to operate through eclipse, helping maintain its thermal stability and continue acquiring dark measurements until 30 October 2012. Starting then, the instrument was powered off going into eclipse every orbit and then powered back on after sunrise in order to maintain sufficient battery power for the spacecraft computer. This resulted in large instrument-temperature excursions, causing the thermal model developed from the dark measurements to be extrapolated outside of the previously measured ranges of temperatures observed. It also prevented subsequent dark measurements, as the spacecraft needed to remain solar-pointed when the instrument was powered on. This power cycling also prevented the continuous, six-hour duration gain calibrations, which were thus extrapolated from values earlier in the mission.

### Daylight-Only Operations (DO-Op) Mode

Starting on 31 July 2013, continued aging of the spacecraft batteries necessitated shutting down the instrument-controlling onboard spacecraft computer during the eclipse portions of each orbit. Aside from a one-week campaign period in late December 2013, no instruments were operational until a new, autonomous operations mode was implemented. This Daylight-Only Operations (DO-Op) mode allowed the spacecraft computer to reboot at sunrise each orbit and then power the instruments on (see Woods et al., 2021). DO-Op mode commenced on 24 February 2014.

The TIM thermal excursions with the DO-Op mode were very similar to those of the power-cycling mode (Section 2.1), but the solar-observation times were more limited during the DO-Op mode for two reasons: i) Some time was lost after sunrise on each orbit to boot the spacecraft computer and then apply power to the instruments; and ii) powering the computer off during eclipses results in the loss of acquired data, so measurement data needed to be downloaded either via a ground station overpass or via the Tracking and Data Relay Satellite System near the end of the sunlight portion of each orbit, shortening the effective measurement time before eclipse entry. These resulted in increased measurement uncertainties after the beginning of the DO-Op mode (Section 3.4).

Spacecraft battery brown-outs started subsequently. These did not change operations except to further limit observations and are also included as additional uncertainties.

## Final Calibration Results

Ground and early-mission on-orbit calibrations are described by Kopp, Heuerman, and Lawrence (2005). Many ground-based calibrations are not possible to update from on-orbit measurements but are expected to be constant (such as aperture areas and voltage references) or are accommodated as time-dependent uncertainties to allow for unknown on-orbit effects. The three main TIM calibrations that are performed on orbit include intercomparisons between different cavities to track changes in absorption (due to solar-exposure-caused degradation), periodic calibrations of the servo control-system gains, and dark measurements. The final mission values for these are applied in V.19 data (available from lasp.colorado.edu/home/sorce/data/tsi-data/ and disc.gsfc.nasa.gov/datasets?page=1&keywords=sorce) and described below.

### Degradation Corrections for Cavity Absorption

**Figure 1:**
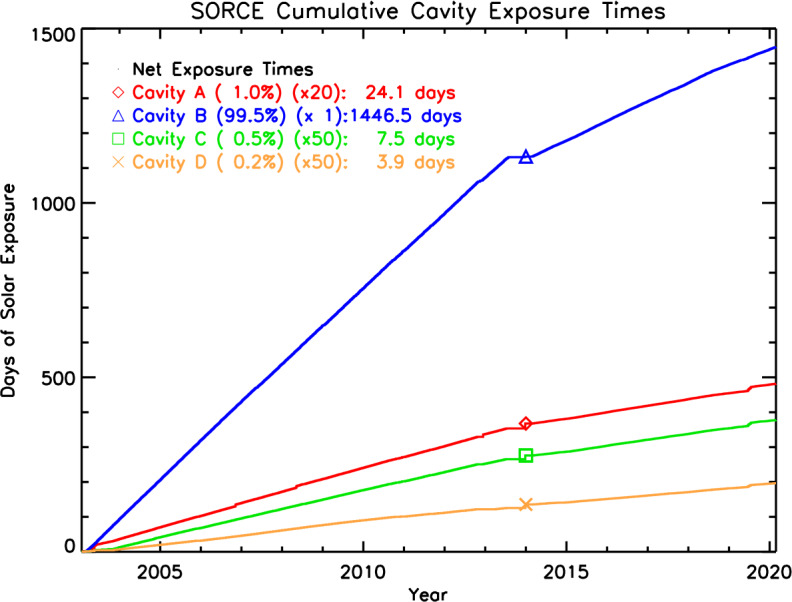
Cumulative exposure times of each cavity are shown throughout the mission. The ratios of exposure rates remained nearly constant with time even after the DO-Op mode started in early 2014. (DO-Op start time is indicated by the symbols plotted on each curve.)

**Figure 2:**
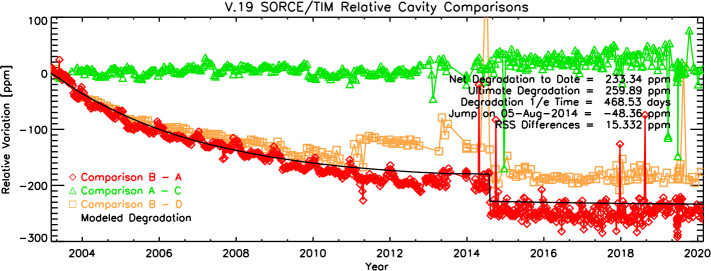
Intercomparisons of the four TIM cavities allow corrections for degradation of the primary cavity, which is the only one to have acquired sufficient solar exposure to show significant efficiency changes over the course of the 17-year mission. This degradation, being about 230 ppm by the end of the mission, is approximately 17 times lower than that of the VIRGO and PREMOS instruments and about 1.5 times lower than in the ACRIM3. The step-function-like jumps in the intercomparisons are not understood and add to Level-3 uncertainties (see Figure 6).

Exposure to unfiltered sunlight causes changes in the black surfaces of the TIM cavities, changing (generally increasing) their reflectances and the associated corrective absorptivity factors applied in data processing. Intermittent and infrequent simultaneous solar observations with pairs of cavities, each having different cumulative solar exposure, allow tracking changes in the primary TIM cavity and enable subsequent corrections in ground processing. Throughout the SORCE mission, the TIM cavity-exposure rates have been 99.5% for the primary cavity (Cavity B), 1% for the secondary (Cavity A), 0.5% for the tertiary (Cavity C), and 0.25% for the quaternary (Cavity D). The rates of exposure of each cavity and the net exposure times are shown in Figure 1. The duty cycling is accomplished by observing for the sunlit portion of one orbit per week with both Cavities B and A, one every other week with Cavities A and C, and one every four weeks with Cavities B and D. As the lesser-used cavities have much lower cumulative solar exposure, their surfaces remain more stable and the relative differences to the primary cavity can be tracked and corrected in ground-based data processing. The results of these cavity intercomparisons throughout the mission are shown in Figure 2.

The primary cavity brightened by 230 ppm by the end of the mission. This is most evident from the cavity intercomparisons between Cavities B and A in Figure 2 and is consistent with comparisons between Cavities B and D. The relative changes show a nearly exponential change with time and are fitted and corrected as such. The secondary cavity shows a very slight ($\approx10~\text{ppm}$) linear increase with time. Although small, this is included in the corrections applied to the comparisons between Cavities B and A.

**Figure 3:**
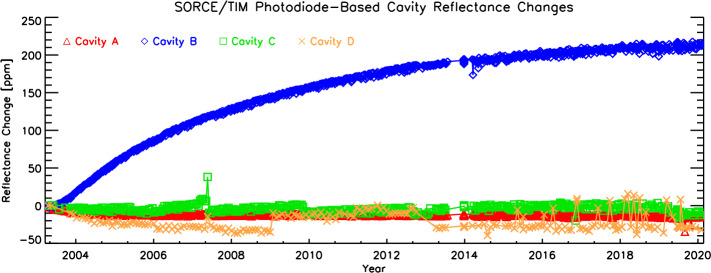
Photodiode signals indicate similar levels of cavity brightening as the cavity intercomparisons, with Cavity B brightening by approximately 220 ppm during the mission.

Four photodiodes, one per cavity, monitor the reflected light from each cavity. These have gains set to measure the reflected sunlight, which is $\approx200~\text{ppm}$ of that incident, so have sufficient sensitivity to detect changes in reflectance of $\approx1~\text{ppm}$. After correcting for common-mode radiation effects in the photodiodes, the resulting estimated reflectance changes are shown in Figure 3. It should be noted that the photodiodes do not sample the cavity interiors uniformly, and thus they cannot be directly used to measure cavity absorptivity changes with time. Instead, they provide a reassuring validation of the direct cavity intercomparisons shown in Figure 2.

The net degradation in the TIM’s primary cavity is approximately 17 times lower than that of the *Solar and Heliospheric Observatory*’s (SOHO’s) *Variability of Solar Irradiance and Gravity Oscillations* (VIRGO: Fröhlich et al., 1995, 1997) and *Picard*’s *PREcision MOnitor Sensor* (PREMOS: Schmutz et al., 2013) TSI instruments, both of which showed approximately a 4000-ppm change in the primary cavity. The *Active Cavity Radiometer* *Irradiance Monitor* (ACRIM3: Willson and Helizon, 1999) showed over 300 ppm of degradation (R. Willson, private communication, 2005), also making the TIM more inherently stable than this newest of the ACRIM series.

### Servo-System Gain Calibrations

**Figure 4:**
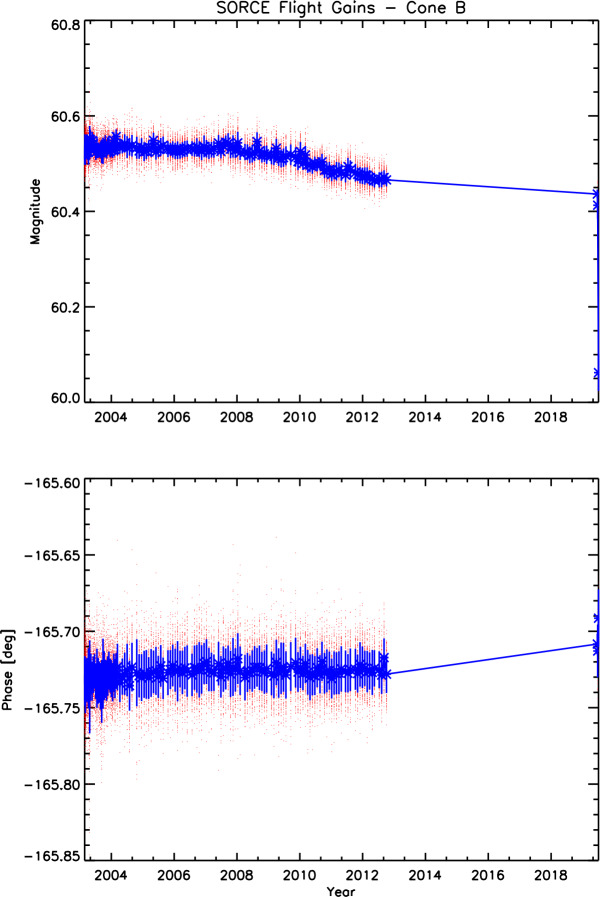
Servo-system gains were calibrated monthly on orbit until the beginning of instrument power cycling. These remained very stable throughout the mission and have nearly negligible contribution to the measurement uncertainties. The red dots are individual high-cadence measurements and the blue error bars give the averages and standard deviations of each monthly calibration.

Servo-system gains are calibrated on orbit by analyzing the cavity thermal-system response to an applied square wave of known amplitude. Both gain magnitude and phase are very stable with time, as shown in Figure 4. (Note the vertical scale.) Nevertheless, this small time-dependence is included in data processing. The instrument is nearly insensitive to this gain value because a feedforward signal is applied to anticipate the needed change in compensating electrical power as sunlight is modulated by a bi-positional (opened/closed) shutter in front of each cavity, so error signals from the servo system are small (see Kopp and Lawrence, 2005). Despite the DO-Op mode, which precluded the six-hour continual gain calibrations that were possible earlier in the mission, attempts were made near the end of the mission to acquire additional gain calibrations. These are also included in Figure 4. Although showing larger deviations from prior in the mission, they do not suggest that changes occurred after the TIM started power cycling, as they were acquired at abnormal instrument temperatures due to the DO-Op mode. The results in Figure 4 are consistent with the data taken prior when accounting for those large thermal variations.

Overall, the instrument gains remained very stable and the effects from the gains have nearly negligible contribution to the TIM measurement uncertainties (see Kopp, Heuerman, and Lawrence, 2005).

### Dark (Thermal Background) Measurements and Model

**Figure 5:**
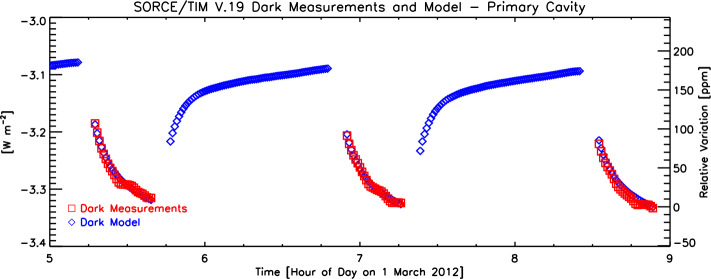
Dark measurements acquired on the eclipsed portions of orbits (red) are fitted to a thermal model (blue) using four instrument temperatures. This allows corrections for the instrument’s thermal background at times of solar measurements, during which direct dark measurements are not feasible.

Thermal background from the instrument contributes approximately a $3.1~\text{W}\,\text{m}^{-2}$ loss to the measured solar signal due to internal instrument radiation being emitted when a cavity’s shutter is open. This is calibrated by looking at dark space during the eclipsed portions of orbits. The emission changes with instrument temperature, so the actual measurements from each cavity are fitted to four instrument thermistors. These sample the stable and temperature-controlled internal heat sink, that cavity’s shutter temperature, the aperture temperature, and a pre-baffle that is directly exposed to the Sun. For each thermistor temperature [$T$], those are fitted as $T^{4}$ to the measured dark-space signals. Since the instrument temperatures are different during the sunlit portion of the orbits due to solar exposure, that model then enables estimating the instrument’s thermal background contribution appropriate for the instrument temperatures during the actual solar measurements. The model fitted to the dark measurements and the calculated values appropriate for the solar measurements are shown in Figure 5. This is applied as a static model throughout the mission, as it was not observed to change during the pre-power-cycling portion of the mission when the dark measurements could be acquired. The model after beginning power cycling was presumed to remain constant, even though that could no longer be verified.

### Time-Dependent Uncertainties

**Figure 6:**
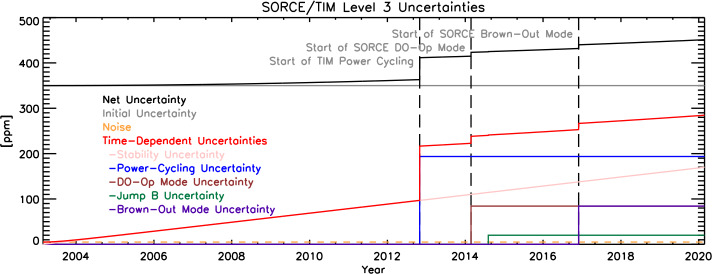
Uncertainties of the Level-3 data as functions of time for various causes.

The SORCE/TIM had an at-launch estimated relative standard uncertainty (“accuracy”) of 350 ppm (see Kopp, Heuerman, and Lawrence, 2005). With time since the final ground calibrations were acquired and subsequent on-orbit operations and possible degradation, knowledge of some of the calibration factors worsens. Estimates of those changes give a temporal dependence to the net uncertainties. Additionally, abrupt changes in operations, such as the large thermal excursions incurred by power cycling every orbit, cause abrupt increases in uncertainties. These are broken out by various contributing factors in Figure 6. The combined time-dependent values are included in the released V.19 data. The end-of-life net uncertainty of the instrument is approximately 460 ppm.

## SORCE/TIM Highlights

The TIM’s estimated beginning-of-mission uncertainty (“accuracy”) was $\approx350~\text{ppm}$, making it ten times better than prior instruments’ actual accuracies. Combined with the TIM’s extremely low noise and best inherent stability of any TSI instrument, the instrument achieved several science breakthroughs. These are summarized in this section.

### Improved Accuracy Established Lower TSI Value

**Figure 7:**
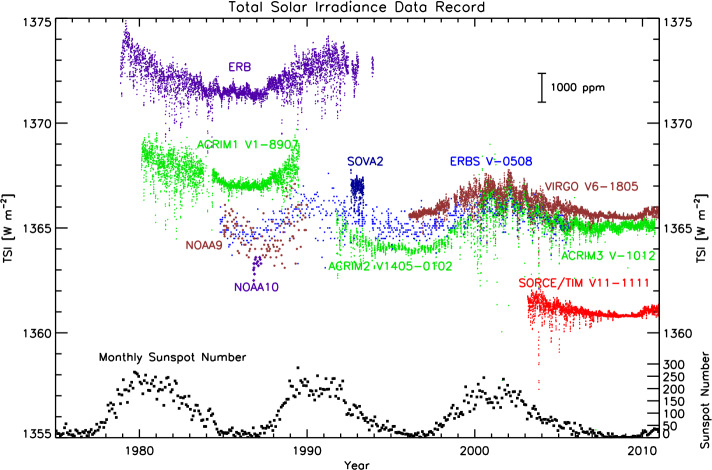
The TSI record at the time of launch of the SORCE mission showed consistency between predecessor instruments near the then-accepted value of $1366~\text{W}\,\text{m}^{-2}$.

At the time of the SORCE launch, the accepted TSI value was $\approx1366~\text{W}\,\text{m}^{-2}$, being the approximate value of the concurrent VIRGO, ACRIM3, and *Earth Radiation Budget Experiment* (ERBE: Lee, Barkstrom, and Cess, 1987) instruments (see Figure 7). The SORCE/TIM, expected to be more accurate with its many intrinsic improvements and latest calibrations, reported TSI values of $1361~\text{W}\,\text{m}^{-2}$, or 0.35% lower. This difference was much greater than the stated uncertainties of the on-orbit instruments, which were 0.035% for the TIM and $\approx0.1\%$ for the other instruments (Kopp, Lawrence, and Rottman, 2005). This unexplained difference persisted for several years while instrument calibrations were checked.

A ground-based version of the TIM verified its optical power measurements directly against the NIST/Gaithersburg Primary Optical Watt Radiometer (POWR). The TSI Radiometer Facility (TRF: Kopp et al., 2007) was built as part of the NASA *Glory* mission. Based on a custom high-power cryogenic radiometer that was calibrated using the POWR, the TRF was the first (and still only) ground-based calibration facility able to calibrate or validate a TSI instrument for irradiance measurements under flight-like conditions of full solar power levels while operating under vacuum. The facility also enabled instrument diagnostics via a high-power incident beam having a programmable spatial profile.

**Figure 8:**
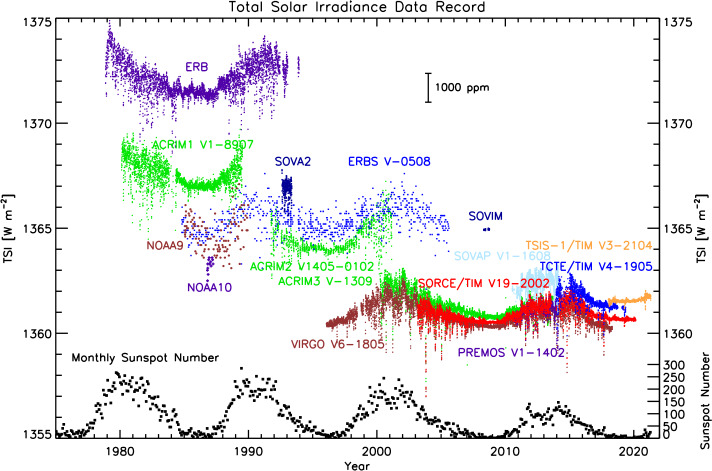
The current TSI record shows good agreement with the TSI value established by the SORCE/TIM from all of the latest instruments.

The new TRF helped resolve the TSI offset issue in two ways: It confirmed the TIM irradiance values; and it indicated high internal-instrument scatter in all tested non-TIM TSI instruments. The high scatter was largely the cause of the higher TSI values reported by the other flight TSI instruments, as explained by Kopp and Lean (2011) as being due to a different optical-aperture configuration in all non-TIM instruments. Subsequent corrections based on these TRF diagnostics were retroactively applied to the flight data of those other instruments; first for the ACRIM3 in 2011 (Willson, 2014) and then the VIRGO in 2014 (Fröhlich, 2014), with both being approximately 0.35% erroneously high prior. The *Picard*/PREMOS, the *TSI Continuity Transfer Experiment* (TCTE)/TIM, and the *Total Spectral and Solar Irradiance Sensor* (TSIS-1)/TIM were all calibrated (for the PREMOS; Schmutz et al., 2013) or validated (for the TCTE and TSIS-1 TIMs) on the TRF prior to launch, and all have transferred that calibration to orbit and confirmed the lower TSI value established by the SORCE/TIM. Current values reported by these and other TSI instruments are shown in Figure 8. Fehlmann et al. (2012) also used TRF calibrations to transfer the facility’s SI-traceability to the World Radiometric Reference (WRR), finding the WRR similarly 0.34% too high on the Système International (SI) scale.

This lower TSI value established by the SORCE/TIM and propagated retroactively to predecessor TSI instruments is now also the currently accepted value by the International Astronomical Union (IAU) (Prša et al., 2016) and thus has been propagated into the Sun’s latest brightness temperature. Loeb et al. (2012) were among the first to use this lower value to help resolve discrepancies between the Earth’s incoming and outgoing radiation, improving uncertainties regarding measurements of the Earth’s energy balance.

### Instrument Sensitivity

**Figure 9:**
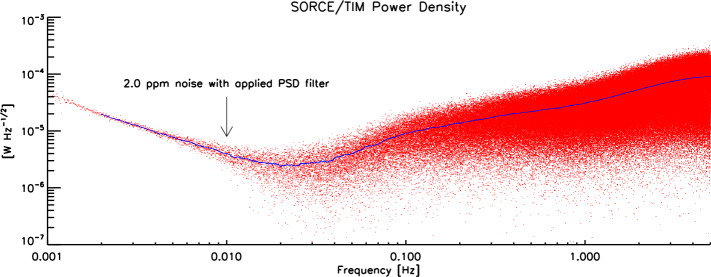
The TIM’s noise power spectrum reaches a minimum near the shutter fundamental of 0.01 Hz, at which point it is 2 ppm. The line (blue) is a smoothed average of the individual power-spectrum points (red).

A phase-sensitive detection (PSD) methodology used in ground-based data processing significantly reduces the TIM sensitivity to noise not in-phase with the instrument’s 100-second shutter period (see Kopp and Lawrence, 2005). On-orbit inter-instrument comparisons during a time of little solar activity near the 2008 solar minimum provided an independent verification that the SORCE/TIM has the lowest noise (and therefore the highest signal-to-noise) of any on-orbit TSI instrument (Kopp, 2014). A power spectrum of the instrument’s on-orbit noise is shown in Figure 9 and gives a measurement noise of 2 ppm with the PSD analysis method.

### Instrument Stability

The TIM’s cavity intercomparisons used to track and correct for on-orbit degradation show a nearly exponential variation with time (which, in turn, is nearly linear with solar exposure) and indicate an end-of-life degradation of 230 ppm (see Figure 2). This is significantly lower than the degradation in other instruments, which is $\approx4800~\text{ppm}$ over 22 years of operations on the SOHO/VIRGO (Fröhlich, 2018), nearly 3000 ppm in two years of operations for the very similar *Picard*/PREMOS (Ball et al., 2016), more than 1000 ppm for the ACRIM2, and greater than 300 ppm for the ACRIM3 (R. Willson, private communication, 2005). While all instruments track these on-orbit changes and correct for them in ground processing, some argument can be made that a smaller correction carries with it a smaller uncertainty, thus favoring a more intrinsically stable instrument.

Such measurement stability is important for tracking long-term changes in the Sun’s variability, as needed for climate studies.

### TSI Record Duration

**Figure 10:**
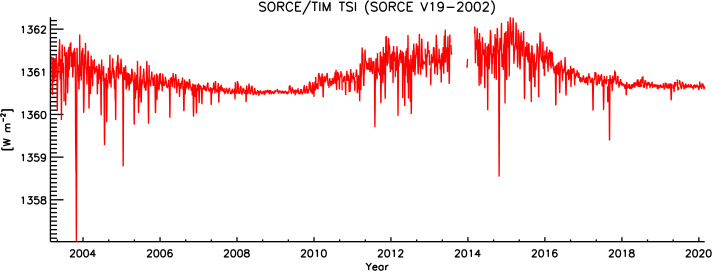
The SORCE/TIM’s measurement record spans 17 years, recording two solar minima and periods of high solar activity near maxima.

The SORCE/TIM achieved exactly 17 years of measurements, spanning from 25 February 2003 to 25 February 2020 (see Figure 10). This duration greatly exceeded the SORCE mission’s five-year-lifetime goal. The TIM thus directly contributes to a significant fraction of the current 43-year spaceborne TSI measurement record. Spanning 1.5 solar cycles, the SORCE/TIM TSI record includes two solar minima as well as a period of very high solar activity in October 2003, when the SORCE/TIM measured the largest short-term decrease in the TSI ever recorded. For climate studies, the longer a high-quality record such as this becomes, the more value it provides.

### Solar Flare(s)

**Figure 11:**
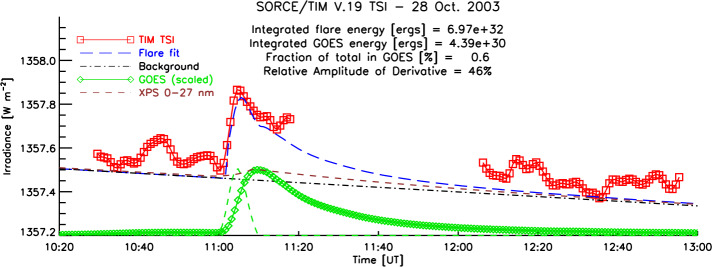
The X17 flare on 28 October 2003 was the first ever observed in TSI (red squares). A fit (dashed blue line) including a linearly varying background (dot–dashed black line) is based on GOES data (green diamonds) and its derivative (dashed green line), which is representative of the impulsive phase of the flare. [Updated data version and plot from that in Woods, Kopp, and Chamberlin, 2006.]

The SORCE/TIM recorded the first solar flare ever observed in the TSI (Woods et al., 2004). This is a result of the instrument’s low noise (Section 4.2) and a great deal of luck to be observing during the daylight portion of the mission’s low-Earth orbit at the time of the flare’s ≈ ten-minute-duration peak. Several other smaller flares were also observed and reported (Woods, Kopp, and Chamberlin, 2006). These detectible flare occurrences are rare, as the spectrally integrated radiant energy from a flare is a very small fraction of the total amount of radiant energy from the entire visible solar surface. The disk-centered X17 flare on 28 October 2003, which was the fourth largest ever recorded by GOES, had a flare peak of a mere 0.027% of the mean TSI value (see Figure 11). The ever-present superposition of globally averaged solar oscillations and convection, for comparison, have amplitudes of 0.005% to 0.01% and occur on comparable five- to ten-minute timescales, so they often mask the signals from smaller flares. Nevertheless, observations of flares in TSI provide the only direct measurement of the net radiant energy released by these large eruptive events.

### Venus and Mercury Transits

**Figure 12:**
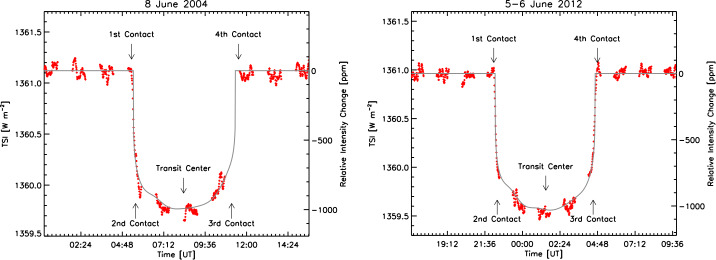
The SORCE/TIM observed two Venus transits across the solar disk. The red dots are the TSI measurements, which closely match the predicted transit light curve (gray line) accounting for solar limb darkening and the SORCE position. The gaps in the plotted data are from times when the SORCE spacecraft was eclipsed by the Earth. The small fluctuations in brightness on short timescales are from normal solar convection and oscillations and can be seen in the un-occulted times both before and after the transits. All data are from SORCE/TIM V.19.

The SORCE observed two Venus transits across the solar disk. These are exceedingly rare, occurring in pairs spaced 8 years apart with those pairs occurring on alternating 105.5- and 121.5-year cycles. During these transits, which occurred on 5 – 6 June 2012 and 8 June 2004, the TIM measured a decrease in the TSI of $\approx0.1\%$, being comparable to that of a medium-sized sunspot and very much in-line with expectations (see Figure 12).

**Figure 13:**
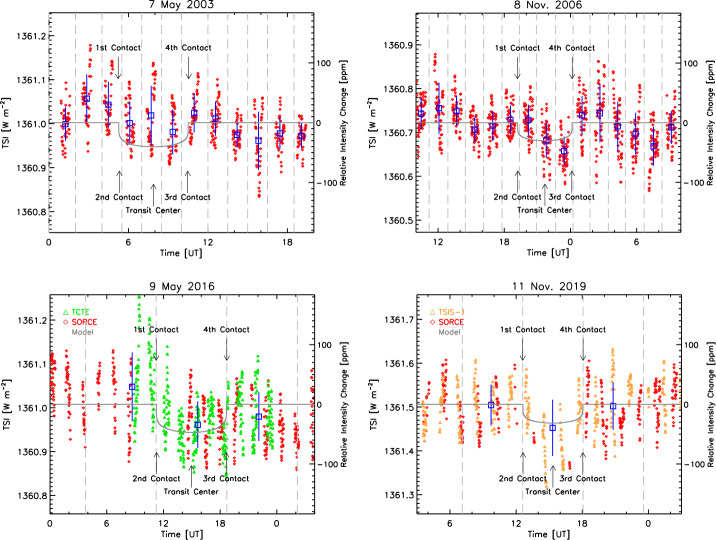
Mercury transits of the solar disk as viewed from the Earth cause $\approx40~\text{ppm}$ decreases in the solar brightness. These signals are largely masked by ever-present solar oscillations and convection, making Mercury-transit detections exceedingly difficult. The blue boxes and whiskers in the latter two transits are, respectively, the averages and standard deviations of equal-length time regions before, during, and after the transit; in the earlier two transits, they are orbital averages and standard deviations. The gray curves are the predicted signals accounting for limb darkening and the spacecraft position. The earlier two transits were viewed only by the SORCE/TIM (red dots), while the 2016 transit includes measurements from the TCTE/TIM (green dots) and the 2019 transit those from the TSIS-1/TIM (orange dots). The SORCE/TIM data are V.19, the TCTE/TIM V.4, and the TSIS-1/TIM V.3.

Exo-solar planets are being discovered via transits in front of their stars using similar photometric techniques by the *Kepler* (Borucki et al., 2010) and *Transiting Exoplanet Survey Satellite* (Ricker et al., 2014) missions. An Earth-like planet transiting a Sun-like star would have a $\approx80~\text{ppm}$ transit depth. Mercury transits of the Sun are good indicators of the expected signals of such exoplanet transits, being roughly 40 ppm in magnitude. The SORCE/TIM observed four such transits (see Figure 13). None of these truly qualifies as an unambiguous “detection,” being largely masked by the greater background signals from solar oscillations and convection, even though some, such as the 2019 transit, look highly suggestive since one knows when to expect the transit. These Mercury transits indicate the level of difficulty in finding an exoplanet that is Earth-like around a Sun-like star.

## Summary

Final (V.19) TSI data products from the SORCE/TIM have been released (available from lasp.colorado.edu/home/sorce/data/tsi-data/ and disc.gsfc.nasa.gov/datasets?page=1&keywords=sorce), completing the instrument’s 17-year measurement record of the net radiant energy incident at the Earth and powering the Earth’s climate system. The many innovations in this new instrument improved the accuracy of the TSI measurement record and propagated that to other TSI instruments, leading to the establishment of the now-accepted TSI value of $1361~\text{W}\,\text{m}^{-2}$. Other science results include the first detection of a solar flare in TSI and the measurements of two Venus and four Mercury planetary transits across the solar disk. The Mercury transits are indicative of expected signals of an Earth-like exoplanet transiting a Sun-like star.
